# The PARP Way to Epigenetic Changes

**DOI:** 10.3390/genes12030446

**Published:** 2021-03-20

**Authors:** Simone Ummarino, Clinton Hausman, Annalisa Di Ruscio

**Affiliations:** 1 Harvard Stem Cell Institute, Harvard Medical School, Boston, MA 02115, USA; 2 Beth Israel Deaconess Medical Center, Department of Medicine, Harvard Medical School, Boston, MA 02215, USA; chausman@bidmc.harvard.edu; 3 Harvard Medical School Initiative for RNA Medicine, Harvard Medical School, Boston, MA 02115, USA; 4 Beth Israel Deaconess Medical Center, Cancer Research Institute, Boston, MA 02215, USA

**Keywords:** genetics, epigenetics, gene regulation, PARP1, ADP-ribosyl transferases, PARylation

## Abstract

ADP-ribosylation, is a reversible post-translational modification implicated in major biological functions. Poly ADP-ribose polymerases (PARP) are specialized enzymes that catalyze the addition of ADP ribose units from “nicotinamide adenine dinucleotide-donor molecules” to their target substrates. This reaction known as PARylation modulates essential cellular processes including DNA damage response, chromatin remodeling, DNA methylation and gene expression. Herein, we discuss emerging roles of PARP1 in chromatin remodeling and epigenetic regulation, focusing on its therapeutic implications for cancer treatment and beyond.

## 1. Introduction

ADP-ribosylation (ADPr) is the most frequent protein post-translational modification (PTM) in eukaryotes. ADPr is mediated by ADP-ribosyl transferases (ADPRT) that covalently attach single or multiple ADP-ribose units from a “*donor*” molecule the nicotinamide adenine dinucleotide (NAD) onto an “*acceptor*” substrate (i.e., proteins, nucleic acids or other small molecules). Poly ADP-ribose polymerases (PARPs) are ADPRT producing chains of ADP-ribose polymers (PAR) of variable sizes (from 2 to more than 200 units) and structures (linear and branched) [1,2] through a process known as *PARylation*. PARylation is involved in a plethora of biological processes that control the cell fate including: chromatin remodeling, DNA methylation changes and gene expression [3,4,5]. The present review discusses the multiple functions of PARP1 in normal and cancer cells [6], expanding on its emerging role as a novel therapeutic target for clinical applications.

## 2. The PARP Family Members

**Figure 1:**
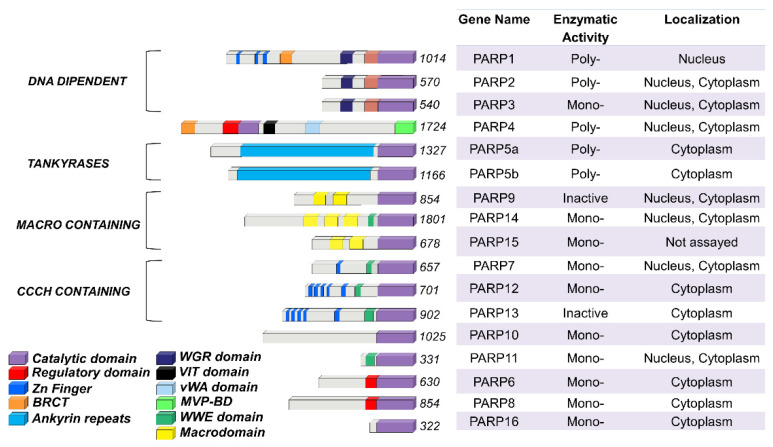
**PARP family members functional domains**. The structures are schematized in colored bars and their specific enzymatic activity including: Mono-, Poly-(ADP)ribose polymerases or Inactive is also reported.

The PARP family consists of seventeen enzymes categorized into four subfamilies and classified according to their structures and domains a representative scheme is shown in Figure 1. 

The DNA-dependent PARPs include PARP1, PARP2, and PARP3. These are activated by discontinuous DNA structures through their amino-terminal DNA-binding domains [7]. The tankyrase subfamily, comprised of tankyrase 1 (also known as PARP5A) and tankyrase 2 (also known as PARP5B), is characterized by ankyrin domain repeats. The ankyrin-domain is a 33-residue motif consisting of two α helices separated by loops. It is responsible for protein protein interactions [8]. The CCCH subfamily contains Cys-Cys-Cys-His zinc-finger motifs that bind the RNA (PARP7, PARP12 and PARP13) and a WWE sequence, consisting of Trp-Trp-Glu domains, possesses PAR-binding activity (PARP14, PARP7, PARP12, PARP13, PARP11). The fourth and final subfamily, macro PARPs, possess ADP-ribose-binding macro domain (PARP9, PARP14, PARP15).

Of the PARP members, PARP1 has been extensively studied and is translated to clinical use. Identified over 50 years ago, PARP1 is the most abundant PARP isoform, localized predominantly in the nucleus [9,10]. The human PARP1 is a 113 KDa protein with a modular structure composed of multiple independent domains: the DNA-binding domain (DBD; residues 1–374), the auto-modification domain (residues 375–525), and the catalytic domain (residues 526–1014). The DBD at the N-terminal end contains two zinc finger motifs (Zn1 and Zn2) that are able to bind to DNA structures [11], nuclear localization signals [12], and a caspase-3 cleavage site [13]. The auto-modification domain includes the typical fold recurrent in DNA repair proteins—a BRCA1 C-terminus (BRCT) arrangement that mediates protein protein interactions and recruits DNA repair enzymes [3]. The catalytic domain of PARP1 at the C-terminal of the primary protein structure is highly conserved in the PARP superfamily, particularly within the NAD binding region [14].

## 3. Basic Notions of PARylation

**Figure 2:**
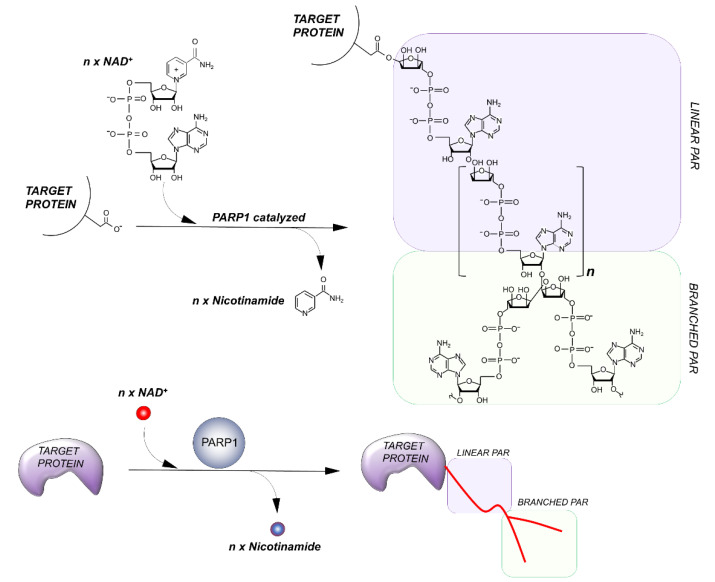
**Outline of the PARylation reaction**. The elongation reaction results in the production of a linear PAR chain; branching instead induces the rising of collateral branches.

PARPs catalyze the transfer of ADP-ribose units from NAD^+^ to specific target proteins, modulating their biological functions. This process, known as PARylation, generates one ADP-ribose and one nicotinamide per molecule of NAD converted. The ADP-ribose unit is then attached on carboxyl group from Glu, Asp and/or Lys residues in the target protein structure [15]. The bond formation between ADP-ribose units during PARylation occurs by either elongation or branching a schematic of the reaction is shown in Figure 2.

During elongation, adenine-proximal ribose units attach to the α (1 → 2) O-glycosidic bond and produce a linear PAR chain. During branching, nicotinamide-proximal ribose units induce the rising of collateral branches. Elongation reactions generate PAR-polymers composed of more than 200 units. Branching reactions occur with less frequency, about once for every 20 elongation reactions [16].

Not all PARP members are capable of carrying out PARylation. Those acting as mono ADP-ribosyl transferases are generally referred as ADP-ribosyl-transferases (ARTDs). Other PARP members lack enzymatic activity entirely. While PARP1, PARP2, vPARP (vault PARP; also known as PARP4), PARP5A, and PARP5B catalyze PARylation, PARP3, PARP10, PARP14 and PARP15 are mono ADP-ribosyl transferases. PARP16 and 17 are catalytically inactive, mediating ADP-ribosylation through interactions with specific yet often unknown cofactors [17,18]. Although features of PARylation have been well defined for PARP1 and PARP2, the variety of mechanisms of other PARP members remains poorly understood.

## 4. Getting Closer to the Edge of Unexplained PARP Functions

**Figure 3:**
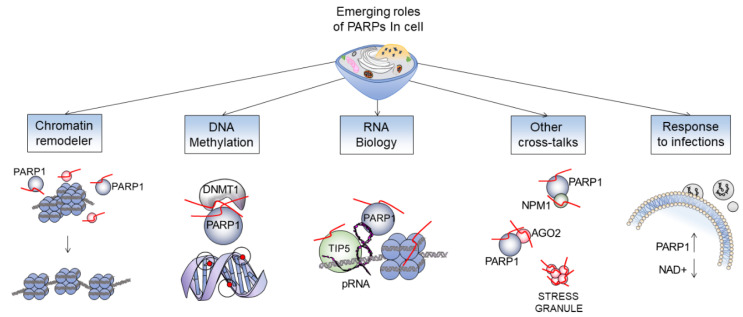
**The wide range of functions carried out by PARP enzymes**. Chromatin remodeling: PARylation determines conformational changes of the chromatin [3]; change in methylation profile: PARP1 neutralize DNMT1 activity [20]; RNA biology: the promoter RNA (pRNA) mediates association of TIP5 and PARP1 and activates the enzymatic activity of PARP1 to PARylate PARP1 itself, TIP5, or histones [21]; other cross-talks: depicted few examples of new emerging interactions of PARP1 with some nuclear proteins; response to infection: the consumption of NAD in cells is dramatically increased by the activation of PARP1 during infections (i.e., COVID 19) [22].

Over the years, the role of PARP enzymes has been primarily investigated in DNA-repair, providing a model to study changes of chromatin structure in response to genotoxic stress. PARP proteins are as now proven targets for new therapeutic approaches in a number of diseases including cancer [19]. Recent studies have clearly illustrated that the range of action of PARP family members covers a plethora of biological processes, including: chromatin and epigenetic remodeling, DNA methylation changes, PARylation-dependent cross-talks and response to infectious diseases as summarized in Figure 3. Yet, many of these mechanisms have remained poorly understood.

## 5. Remodeling of Chromatin: Can Histones Be PARylated?

Is PARP1 able to induce chromatin structure modifications? The first attempt to answer this question was made in 1983 by Aubin et al. [23]. Quenet et al. [24] later clarified that PARP1-mediated PARylation can activate chromatin compaction and condensation. In vitro evidence has shown that the histone core of nucleosomes (H2A, H2B, H3, and H4) as well as the linker histone H1, could be PARylated by PARP1. This discovery led to hypothesize the involvement of PARylation in chromatin decondensation [25]. However, the mechanism of PARP1-induced histone chromatin rearrangement might be more sophisticated. From a structural prospective, it is reasonable to assume that the addition of highly negatively charged PARs, wrapped around chromatin proteins, should repel the nearby DNA thereby inducing chromatin decondensation.

Few studies have focused on the interplay between PARP1, PARylation, and histones. Kim et al. (2004) demonstrated that PARP1 binds to the linker DNA [26], and that this particular site corresponds to the H1 localization. Consequently, PARP1 and H1 compete for a binding to nucleosomes in vitro [25]. Further studies have proved that competition between PARP1 and H1 to targeted gene promoters does contribute to the dynamic regulation of gene expression [27,28]. However, while PARP1 preferentially associates with less condensed chromatin, H1 mainly co-localizes with highly condensed chromatin.

In terms of DNA repair, PARP1 has been implicated in the removal of histones near DNA lesions to allow for the translocation of DNA damage response (DDR) enzymes. A 2020 study found that ADP-ribosylation was associated with histones removal from DNA lesion sites. Consistently, treatment with a PARP inhibitor prevented the eviction of histones at DNA lesion sites [29].

Interestingly, PARP1 was also found to interact with the H2A variants H2A.Z and macro H2A. These interactions might lead to recruitment and integration of histone variants to specific genomic sites and control PARP1 function [30,31]. The PARP1 automodification domain seems to promote PARP1 release from DNA and/or convert PARP1 into a histone-binding protein [32].

Consistent with this study, Gibbs-Seymour et al. discovered a novel protein, the histone PARylation factor 1 (HPF1, or C4orf27) as a PARP1-interacting component of double strand break (DSB) repair. In human cells, HPF1 is capable of regulating the PARP1 automodification domain, thus promoting ADP-ribosylation of the histones to ensure genomic stability [33].

Interestingly, PARP1 is also able to directly PARylate DNA breaks by loading PAR units to the terminal phosphates, as recently shown by Matta E. et al. [34].

## 6. PARP1 as an Effector of Chromatin Modifications

PARylation is implicated in modulating the activity of histone and chromatin modifying enzymes at the different chromatin levels:

***a.***  ***Constitutive heterochromatin***, highly condensed regions of DNA that display species-specific genomic coverage and variability ranging from 30% to 90%;
***b.***  ***Facultative heterochromatin***, regions of packaged DNA that can be reverted to euchromatin upon specific conditions and histone modifications, accounting for 45% of the genome;
***c.***  ***Euchromatin***, highly accessible and decondensed portions of the DNA that are transcriptionally active.

**Figure 4:**
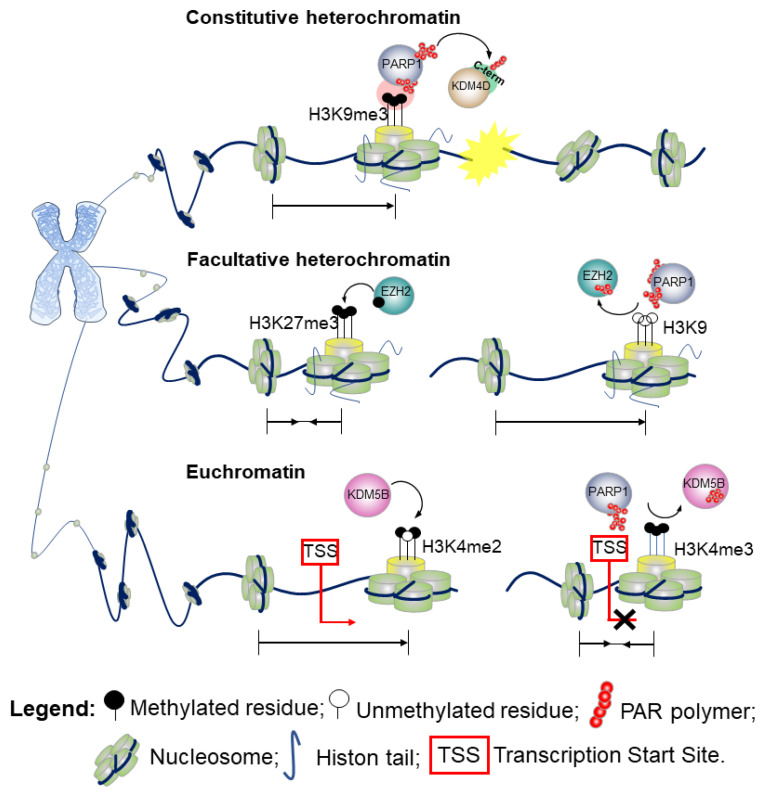
**PARP1 plays a role in chromatin modifications**: euchromatin, constitutive heterochromatin, and facultative heterochromatin.

**a.**  ***Constitutive heterochromatin***
  H3 lysine 9 trimethylation (H3K9me3) is the hallmark of highly condensed chromatin. Defects in PARylation are commonly associated with loss of the methylation marker at the centromeric heterochromatin of pericentromeric regions. While the di-(me2) and tri-methylation forms of H3K9 are enriched at the transcriptional start site (TSS) of silenced genes, the mono-methylation variant (H3K9me1) marks promoters of actively transcribed genes [35]. Although H3K9me3 aids the recruitment of chromatin enzymes involved in the DDR, its presence impairs the DNA repairing process that requires a decondensed state of chromatin to enable the action of the DNA-repair effectors [36]. PARylation of the lysine-specific demethylase 4D (KDM4D) [37] at the C-terminal domain engages KDM4D to the sites of DNA damage. By promoting the demethylation of H3K9, PARylation reduces the degree of chromatin compaction, thus playing a key role in the propagation of DDR in vivo [37,38]. A schematic outlining this molecular mechanism is shown in Figure 4 (upper part). Further evidence show that in response to DNA damage when the chromatin undergoes structural reorganization to ensure accurate DNA repair [3], PARP1 not only promotes recruitment of proteins at the damaged site, but also acts as a chromatin remodeler to facilitate the access of the DNA repair machinery. Indeed, several studies have demonstrated that PARylation of the histones causes chromatin decondensation [25,39].
  For instance, by recruiting chromodomain helicase DNA binding protein 2 (CHD2) at DSBs, PARP1 triggers deposition of the histone variant H3.3, and ultimately chromatin relaxation thereby regulating the assembly of non-homologous end-joining (NHEJ) complexes to rescue genomic integrity.
  Therefore, PARP1 links CHD2-mediated chromatin expansion and H3.3 deposition to DNA repair by NEHJ. In the NHEJ pathway, PARP1 may also serve as a scaffold to recruit at sites of DNA damage a number of transcription repression complexes, including the nucleosome remodeler and deacetylase (NuRD), the complex proteins CHD4, the metastasis-associated protein 1 (MTA1) [40,41], and members of Polycomb repressive complex 1 (PRC1) [40].
**b.**  ***Facultative heterochromatin***
  A positional effect of PARylation has also been proposed in the context of facultative heterochromatin as observed for the H3K9me3/2 demethylase KDM4D [42]. Indeed, PARylation of KDM4D conserved N-terminal domain—the JmjN, that is a substrates for PARP-1, inhibits its activity at the promoter of retinoic acid receptor (RAR)-dependent genes thereby resulting in transcriptional repression [42]. Hence, whilst PARylation at the C-terminal domain promotes KDM4D demethylase activity and reduces the degree of chromatin compaction [37], PARylation at the N-terminal end results in an opposite effect. Alternatively, a model wherein PARP1 cooperates in the establishment of the heterochromatin landscape upon inhibition of KDM4D has also been proposed [42]. This action of PARP1 can be reversed by poly-ADP-ribose glycohydrolase (PARG), the catabolic enzyme that cleaves the ADP-ribose polymers synthesized by PARP1. PARG counteracts the action of PARP1 and favors an open structure of the chromatin, promoting an active transcriptional state [42].
  In the attempt to further understand the PARP1 and PARylation conundrum, a 2015 study investigated the effects of PARP1 on global gene expression in a lymphoblastoid B cell line [43]. These data revealed that PARylation controls the methyltransferase enhancer of zeste homolog 2 (EZH2), the catalytic subunit of the polycomb repressive complex 2 (PRC2). PRC2 is responsible for the trimethylation of the lysine 27 on histone 3 (H3K27me3), which leads to chromatin compaction and gene silencing. A schematic example of the molecular mechanism is presented in Figure 4 (middle part).
  Upon pharmacological inhibition of PARP and shRNA-mediated downregulation of PARP1, EZH2 expression is induced, resulting in increased global H3K27me3 [43].
  Furthermore, PARP activity is required for retaining PRC2, the supporting protein suppressor of Zeste 12 (SUZ12) and the embryonic ectoderm development (EED), at the site of DNA damage. Surprisingly, EZH2 is not recruited directly by single-strand breaks or UV damage [44].
**c.**  **Euchromatin**
  Two methylation states of H3 lysine 4 (H3K4me2/me3) are enriched at the TSS of actively transcribed genes and correspond to euchromatic regions in the genome. The monomethylation state typically marks enhancers [45]. As shown in Figure 4 (bottom part), PARylation impairs the enzymatic activity of KDM5B, a histone lysine demethylase of the H3 trimethylated lysine 4, and the respective binding to H3 in in vitro assays. Consistently, inhibition of PARylation in vivo results in increased levels of KDM5B at the TSS of active genes and decreased levels of H3K4me3.
  The interplay between PARP1 and KDM5B has been considered a regulatory mechanism to control the chromatin state at the basal and signal-transcriptional level [27]. While PARylation recruits KDM5B to DNA damaged sites, demethylation of H3K4me3 in proximity to DNA breaks helps to recruit proteins involved in the DNA-damage repair, including BRCA1 [46]. Hence, PARylation of KDM5B could have a double effect on chromatin association. Remodeling of the chromatin during DSB repair can include variation on the usual mechanism observed in gene transcription, including the physical movement of nucleosomes, histone variant exchange, and dynamic changes in histone acetylation and methylation to create nucleosome-free regions that facilitate the entire repair process [47]. A recent finding from Gong et al. using live imaging, revealed that PARP1 recruits KDM5A through PAR chains at the damaged chromatin side, leading to rapid erasure of H3K4me3 and promoting recruitment of a second repair protein, ZMYND8 [48]. Consistent with these findings, loss of KDM5A attenuates the normal drop in local transcriptional activity adjacent to DSBs, in line with loading of ZMYND8 (and loss of H3K4me3) acting as a general transcriptional repressor [48].
  Further, PARP1–3 proteins can directly PARylate DNA breaks by loading PAR units to terminal phosphates. [34].
  Finally, a 2019 study details a fascinating interplay between PARP1, chromatin, and RNA polymerase II (RNAPII). It was found that RNAPII pauses elongation when it encounters PARP1 bound to chromatin. Knockout of the PARP1 gene prevented this pause from occurring, implicating that PARP1 plays a regulatory role in chromatin changes and transcription [49].

## 7. PARP1 Modulates the Delicate Balance of DNA Methylation

DNA methylation is the major epigenetic modification in eukaryotic genomes. It occurs at position 5 of cytosine when followed by guanosine (CpG) in mammals. In the human genome, methylated cytosines (5mC) are mainly clustered in discrete regions termed CpG islands (CGIs), which account for 1% of the whole genome. CGIs are located in the vicinity of TSSs in the majority (~70%) of protein coding genes. While the bulk of genome is methylated at 70–80% of its CpGs, CGIs are mostly unmethylated in somatic cells and are transcriptionally permissive [50]. DNA methylation is catalyzed by three DNA methyltransferases which have different roles in maintenance (DNMT1), and de novo methylation (DNMT3a and DNMT3b). DNMT1, the main mammalian DNA methyltransferase, is localized in the replication foci and is responsible for copying methylation patterns after DNA replication [51]. The DNA methylation profile is established during cell development and differentiation. Its importance is proven by the lethal phenotypes resulting from DNMTs’ knock-out in vivo models (Manzo et al., Embo J, 2017) [52]. Furthermore, aberrant DNA methylation remains a hallmark of cancer progression [53] and silencing of tumor suppressor genes [54,55].

The interactions with various proteins or molecules may alter DNMTs’ activity. Over the past decades, Caiafa et al. have provided evidence linking PARylation to DNA methylation. Their data indicate that blockage of PARylation increases DNA methylation levels in vivo, while activation of PARylation is responsible for maintaining the unmethylated status of specific CpGs [56,57,58]. The authors demonstrated that ADP-ribosylated PARP1 isoform is associated with DNMT1 in vivo [20,59,60], which suggests a connection between PARylation and changes in the DNA methylation profile [5,60]. Therefore, the establishment of an epigenetic mark, such as DNA methylation, in normal and cancer cells might occur through PARP1’s regulation. Interestingly, the effects of PARP1 on DNA methylation may be modulated by CCCTC-binding factor (CTCF), which promotes the auto-modification of PARP1 [58,61,62] and is responsible for the cross-talk between PARylated PARP1 and DNMT1 [58,63]. It has also been demonstrated that PARP1 maintains the unmethylated status of specific CTCF-bound CpGs by inhibiting DNMT1’s activity [58]. Genome-wide association studies in breast cancer cell lines showed that PARP1 correlates with other epigenetic elements such as active histone marks [28]. This association is mutually exclusive with DNA methylation and pharmacological inhibition of PARP1 leads to changes in the DNA methylation profile, thus proving a functional interplay between PARP1 and DNA methylation [64].

A role for PARP1 in DNA methylation events involved in cell reprogramming and induced-pluripotent stem cells (iPSCs) has recently emerged. Somatic cells can be reprogrammed into iPSCs by means of four pluripotent transcription factors: Oct4, Klf4, Sox2, and c-Myc, altogether referred to as OSKM [65,66]. PARP1 plays a key role in this mechanism. Four days after OSKM’s transduction, PARP1, together with the ten-eleven translocation-2 methyl-cytosine dioxygenase (Tet2), is recruited at specific loci promoting early epigenetic modifications that are essential for cells reprogramming [67]. That would also explain the high levels of PARs associated with the global demethylation process during reprogramming towards totipotency or pluripotency [68].

## 8. A PARP1 RNA Interplay

The role in RNA biology is emerging as a novel and intriguing function of PARP1. Far from being well understood, PARP1 has been implicated in maintaining ribosomal DNA (rDNA) across cell division. During S phase, PARP1 binds to TTF-1-interacting protein-5 (TIP5), which is part of the nucleolar remodeling complex (NoRC). Promoter-associated RNAs (pRNAs) also bind to this TIP5-PARP1 complex, and the TIP5-PARP1-pRNA complex binds to rDNA. pRNA activates PARP1 enzymatic activity, causing it to PARylate itself, histones, and TIP5 (cite 22617384). This PARylation ensures silenced transcription and the formation of silent rDNA [21].

Although it is known that PARP1 does bind to transcripts originating from an RNA polymerase I (Pol I) promoter located 2 kb upstream of the pre-rRNA transcription start site termed pRNA [69,70], the mechanism of the interaction is mostly unknown. A 2017 study found that PARP1 preferentially binds to RNAs with GC rich regions. The study also found that removal of the Zn1 and Zn2 domains of PARP1 causes the protein to preferentially bind to RNA instead of DNA [71].

PARP1 interaction with long noncoding RNAs (lncRNAs) seems to play a role in pediatric neuroblastomas. For instance, Forkhead box D3 antisense RNA 1 (FOXD3-AS1) is a lncRNA downregulated in neuroblastomas. In the nucleus, FOXD3-AS1 inhibits PARylation by PARP1, causing increased expression of various tumor suppressor genes [72]. FOXD3-AS1 expression is reduced in neuroblastomas, thus causing a decreased expression of tumor suppressor genes by a PARP1-dependent mechanism. Administration of FOXD3-AS1 in neuroblastoma cells results in re-expression of tumor suppressor genes and improved outcomes in murine studies [72].

In addition, other PARPs family members also play interesting roles in RNA regulation. Cellular stress causes the buildup of RNA-rich granules in the cytoplasm. These granules contain six PARPs (PARP5a, PARP12, PARP13.1, PARP13.2, PARP14, and PARP15) and PARs, which regulate mRNA stability and translation [73]. Lastly, RNA-binding PARPs can directly modulate transcription, and PARylation of RNA-modulating enzymes can affect RNA localization, binding, and activity during stress and non-stress conditions [74].

These findings are revealing a critical and fascinating role for PARP1 and other PARPs in RNA biology, that remains to be investigated.

## 9. Mechanisms and Clinical Applications of PARP Inhibitors

PARP enzymes have been shown to play a significant role in DDR by recruiting and PARylating various enzymes. PARP inhibitors are nicotinamide analogs that function by competitively binding to the NAD^+^ binding site on both PARP1 and PARP2 [75] A list of PARP inhibitors is reported in Table 1. Due to PARP’s role in DDR, these inhibitors can find clinical application to increase cytotoxicity in malignant cells by decreasing their ability to repair damaged DNA [76]. PARP inhibitors have been particularly successful in treating germline BRCA mutated cancers [75]. As of January 2021, there are four Food and Drug Administration (FDA) approved PARP inhibitors recommended for the treatment of various cancers (Table 1). A fifth drug, veliparib, is currently in Phase III clinical trials and is showing promising results [75].

PARP inhibitors are most often used in combination with other targeted therapies and chemotherapy. Rucaparib, for example, is commonly used as a maintenance therapeutic alongside platinum-based chemotherapy (cite 30830551). A January 2021 study found that olaparib treatment coupled with stimulator of interferon genes (STING) agonism induces more significant STING activation than STING agonism alone [81]. By increasing cytotoxic T cell response, this combined therapy improved anti-tumor effects significantly [81]. Furthermore, this effect is more significant when also coupled with checkpoint inhibitors that block PD-1 [82].

Similarly, olaparib is used in accordance with chemotherapy and radiation. Interestingly, olaparib’s cytotoxic effects potentiate the effects of chemo and radiation therapy [83]. PARP inhibition is far more cytotoxic than simple knockout of PARP genes [84]. This increase in cytotoxicity can be explained via a process known as “*trapping*”. Through an unknown mechanism, PARP inhibitors lock PARP1 and PARP2 at the site of the damaged DNA [84]. These *trapped enzymes* further prevent other DDR enzymes from translocating to the damaged DNA, further increasing cytotoxicity. Alongside the decrease in catalytic activity, PARP inhibitors exhibit a two-sided attack on malignant cells.

These effects also make the targeted cells more susceptible to chemotherapy and radiation therapy. Alongside the decrease in catalytic activity, PARP inhibitors exhibit a maintenance of heterochromatin throughout cell divisions by controlling UHRF1-DNMT1 interplay and by directing DNMT1 to euchromatin regions and hemi-methylated CpG dyads [79]. The interaction of UHRF1-PARP1 seems also essential for cell viability, as recent findings suggest its involvement in response to DNA damage [80].

Though extremely effective, PARP inhibitor resistance is common and arises via a multitude of mechanisms. BRCA deficient malignancies develop resistance via restoration of homologous recombination (HR) repair [85]. Resistance can also develop through stabilization of replication forks [85]. In the case of rucaparib, sensitivity to the drug in high-grade serous ovarian carcinoma (HGSOC) is determined by the methylation of the BRCA gene. Homozygous methylation predicts improved response to the drug, whereas hemizygous methylation correlates to resistance [86].

## 10. Other Cross-Talks in the Complex Network Created by PARP1

PARPs are involved in several cross-talks with nuclear proteins; however, the way in which many of these interactions occur remains unknown. PARP1 and PARP2 have nuclear and nucleolar localization signals which allow them to localize in both nucleus and nucleolus, respectively. In the nucleolus, through their N-terminal domain, PARPs interact with nucleophosmin 1 (NPM1, also known as B23), a shuttling protein mainly confined inside the nucleolus and involved in ribosomal RNA biogenesis [87,88]. Mutations at PARP’s N-terminal domain prevent the interaction with NPM1. The lack of said interaction does not impair either proteins’ functions [89]. However, it is not clear yet how the PARP-NPM1 complex may act within the cell.

The presence of a hexanucleotide-repeat expansion, composed of 4 guanines and 2 cytosines (GGGGCC), [90,91] within the C9orf72 locus in amyotrophic lateral sclerosis (ALS) has been shown to induce nucleolar stress and DNA damage in motor neurons. PARPs and NPM1 are both involved in DDR by maintaining DNA integrity and recruiting proteins of the DNA base excision repair (BER) system. Given NPM1 is a histone chaperone induced by DNA damage [92], the presence of both PARP1 and NPM1 might be necessary for DNA repair mechanisms. In fact, motor neurons from ALS patients show an up-regulation of DDR markers, including the phosphorylated form of histone 2AX (γ-H2AX) and ataxia telangiectasia mutated gene (p-ATM), the cleaved PARP1, the tumor suppressor p53‑binding protein (53BP1) and other hallmarks of DDR [93]. In this scenario, NPM1 plays a pivotal role in DDR and its overexpression inhibits apoptosis and restores the structure of the nucleolus.

Although poorly characterized, the interplay between PARP1 and NPM1 was shown to be functional for treatment of acute myeloid leukemia. Inhibition of PARP proteins might exert a lethal effect on AML cell lines by interfering with the PARP1/NPM1 interaction [94]. Another interesting aspect of PARylation involves the tumor suppressor 53BP1. 53BP1 has a major role in the NHEJ pathway of DNA repair. When human cells age however the recruitment of the protein to the site of damage is impaired [95]. Recent findings have now revealed that ADP-ribosylation of 53BP1B which is increased in response to DNA damage, is targeted by a PAR-binding E3 ubiquitin ligase, RNF146, leading to 53BP1 ubiquitination and degradation, thus preventing the recruitment of 53BP1B to the DNA damage site. Removal of ADP-ribosylation by the Nudix hydrolase NUDT16 from 53BP, improves 53BP1 stability, prevents the protein degradation and restores localization at the DSBs [96].

Another partner of PARP1 is the E3 ubiquitin-protein ligase UHRF1 [97]. UHRF1 is a DNMT1-interacting protein involved in maintenance of CpG methylation. PARP1 mediates stabilization of the DNMT1-UHRF1 complex. PARylation seems to be required for the maintenance of heterochromatin throughout cell divisions by controlling UHFR1-DNMT1 interplay and by directing DNMT1 to euchromatin regions and hemi-methylated CpG dyads [97]. The interaction of UHRF1-PARP1 seems also essential for cell viability, as recent findings suggest its involvement in response to DNA damage [98].

Cytosolic PARylation catalyzed by some members of the PARP family still remains a largely unexplored mechanism. A hypothesized function foresees the recruitment of RNA-binding proteins to specific loci in the cytoplasm, such as the stress granules (SGs) [73]. Indeed, five out of seventeen PARPs have been identified in SGs including tankyrase PARP-5a, RNA-binding PARP12, PARP13.1 and isoform-13.2, PARP-15. This mechanism resembles the role of pADPr in other cell compartments, such as the recruitment of DNA repair proteins at DNA damage sites into the nucleus or acid-binding proteins for chromatin remodeling [99,100]. Other stress granule proteins including Ago2, G3BP1, and TIA-1 are modified by PARPs and such alterations increases upon cell stress [73]. In light of these data, PARylation may act as a PTM involved in the assembly of cytoplasmic stress granules that accumulates RNA-binding proteins involved in the translation and stability of mRNAs upon stress. In this sense, poly ADP-ribosylation could emerge as a key regulator factor of post-transcriptional gene expression in the cytoplasm [101,102]

## 11. Beyond Cancer Treatment: A Novel Target for COVID-19?

While COVID-19 has been spreading rapidly throughout the world, some research groups are now exploring PARP1 as a possible target for therapeutics. It has been known for over 20 years that PARP1 becomes activated during acute lung injury (ALI), that resembles one of the major complications caused by SARS-CoV-2 [103]. This upregulation is caused by the aryl hydrocarbon receptor (AhR), which is overexpressed in coronaviruses. The AhR regulates PARP1 gene expression, implying that upregulation of PARP1 is likely to result from coronavirus infection [22]. Activation of PARP1 leads to cell death by consuming large amounts of NAD^+^ and ATP; this is even more likely to happen during an infected state [104]. In this state of depleted nutrients, cell death leads to further recruitment and activation of immune cells. This vicious cycle of nutrient depletion and inflammation worsens ALI significantly. Thus, inhibiting PARP1 may be a viable treatment for ALI patients with COVID-19. Murine and early clinical trials have found that PARP1 inhibitors decrease levels of IL-1, IL-6, and TNF-α, which are key interleukins in the cytokine storm caused by COVID-19. Reduction of these interleukins alleviates post-infection lung fibrosis. Evidence also suggests that PARP1 inhibition causes macrophages to become more tolerogenic, further decreasing inflammation [105].At this time, the aforementioned PARP inhibitors have been approved only for cancer therapy and not for pulmonary damage caused by infection. However, it is believed that any of these inhibitors could be effective in treating ALI caused by COVID-19.

PARP1 is not the only targeted PARP for COVID-19 treatment. PARP 7, PARP10, PARP12, and PARP14 are also potential therapeutic targets [105]. As we learn more about the virus, its infection, and lasting consequences, PARP enzymes may provide effective treatment opportunities.

## 12. Conclusions

More than 50 years of research demonstrated that PARP inhibitors are efficient anticancer agents for ovarian and breast cancers. Accumulating evidence suggests that PARP1-mediated PARylation plays a fundamental role in major epigenetic pathways ranging from histone modifications and rearrangements to DNA methylation changes. Yet, a detail characterization of the PARP1-related molecular mechanisms engaged in epigenetic pathways is far from being completely understood.

Recent studies have been trying to dissect the “*modus operandi*” of PARP1 in normal and diseased cells. It has become evident that activation of PARP1 is not solely triggered by DNA damage response in BRCA-1 and BRCA-2 mutated tumors but a number of proteins intervene at different levels by stimulating or inhibiting PARP1 catalytic activity and targeting PAR length. Thus, PARylation should not only be addressed as a target for diseased conditions, but as an important mediator of molecular mechanisms that secure the proper configuration of epigenetic marks.

The present review provides an integrated perspective of underexplored cross-talks between PARP1 and other interacting proteins, critical in the dynamic changes of chromatin conformation, histone PARylation and DNA methylation.

In summary, the many roles of PARP1 poses a plethora of opportunities for targeted therapeutics. Herein, we reviewed details of the latest knowledge in PARP1 research. From cancers to respiratory diseases, PARP1 offers alternative approaches for the development of novel therapeutic strategies.

## Figures and Tables

**Table 1:** PARP inhibitors and their clinical uses.

Name	Description	Reference
**Olaparib**	In HER-2 negative metastatic breast cancer patients with a germline BRCA mutation, olaparib has been shown to be very effective. Response rate of 59.9% compared to 28.8% in the standard therapy group.	[77].
**Niraparib**	In patients with platinum sensitive recurrent ovarian cancer, niraparib greatly enhanced progression-free survival as compared to placebo. These results were consistent regardless of a germline BRCA mutation or homologous recombination deficiency (HRD) status.	[78]
**Rucaparib**	Rucaparib is generally a third (or later) line treatment used in patients with BRCA mutated ovarian cancer and as maintenance therapy for patients with recurrent or relapsed platinum sensitive ovarian cancer. Analysis has revealed an objective response rate of 54%.	[79]
**Talazoparib**	Used in patients with advanced breast cancer and germline BRCA mutations. Talazoparib has shown a significantly higher likelihood of progression-free survival (62.6% compared to 27.2% in the standard therapy group).	[80]

## Data Availability

Not applicable.
